# The Reproductive Success of *Triturus ivanbureschi* × *T. macedonicus* F_1_ Hybrid Females (Amphibia: Salamandridae)

**DOI:** 10.3390/ani12040443

**Published:** 2022-02-12

**Authors:** Tijana Vučić, Ana Ivanović, Maja Ajduković, Nikola Bajler, Milena Cvijanović

**Affiliations:** 1 Faculty of Biology, Institute of Zoology, University of Belgrade, Studentski trg 16, 11000 Belgrade, Serbia; ana@bio.bg.ac.rs (A.I.); nikola.bajler@outlook.com (N.B.); 2 Department of Evolutionary Biology, Institute for Biological Research “Siniša Stanković”, National Institute of the Republic of Serbia, University of Belgrade, Bulevar Despota Stefana 142, 11000 Belgrade, Serbia; maja.ajdukovic@ibiss.bg.ac.rs

**Keywords:** egg size, hybrid breakdown, life-history traits, newts, mtDNA introgression

## Abstract

**Simple Summary:**

Two moderately related large-bodied newt species endemic to the Balkan Peninsula, the Balkan crested newt (*Triturus ivanbureschi*) and the Macedonian crested newt (*T. macedonicus*), coexist and hybridize in central Serbia. Many generations of mutual hybrid crossings and backcrossings with parental species shaped the genetic composition of hybrid populations. Natural populations have admixed nuclear DNA (nuDNA) of parental species and *T. ivanbureschi* mitochondrial DNA (mtDNA), which is usually maternally inherited. The mechanisms that direct gene flow and shape the first generations of hybrids could explain the formation of hybrid zones and their maintenance in nature. We followed and compared life history traits related to reproduction of the first generation of reciprocal hybrids obtained by experimental crossing. Our results suggested that possible incompatibilities between mitochondrial and nuclear genomes, which could lead to the exclusion of *T. macedonicus* mtDNA in natural populations, most likely act at later stages of development or subsequent hybrid generations. Results from this study add to the growing knowledge of *Triturus* hybrid biology and ecology, which is the baseline for conservation programs necessary to protect these highly endangered amphibians.

**Abstract:**

Two large-bodied newt species, *Triturus ivanbureschi* and *T. macedonicus*, hybridize in nature across the Balkan Peninsula. Consequences of hybridization upon secondary contact of two species include species displacement and asymmetrical introgression of *T. ivanbureschi* mtDNA. We set an experimental reciprocal cross of parental species and obtained two genotypes of F_1_ hybrids (with *T. ivanbureschi* or *T. macedonicus* mtDNA). When hybrids attained sexual maturity, they were engaged in mutual crossings and backcrossing with parental species. We followed reproductive traits over two successive years. Our main aim was to explore the reproductive success of F_1_ females carrying different parental mtDNA. Additionally, we tested for differences in reproductive success within female genotypes depending on the crossing with various male genotypes (hybrids or parental species). Both female genotypes had similar oviposition periods, number of laid eggs and hatched larvae but different body and egg sizes. Overall reproductive success (percentage of egg-laying females and viability of embryos) was similar for both genotypes. The type of crossing led to some differences in reproductive success within female genotypes. The obtained results suggest that processes that led to exclusion of *T. macedonicus* mtDNA in natural populations may be related to the survival at postembryonic stages of F_2_ generation or reproductive barriers that emerged in subsequent hybrid generations.

## 1. Introduction

The reproduction of genetically divergent taxa is a frequent phenomenon in natural populations (e.g., [1,2]). Hybrids could be sterile or produce further generations by mutual crossings and/or backcrossing with parental genotypes. The novel combination of genotypes in hybrids could be adaptive and beneficial for fitness, leading to hybrid speciation or these combinations could cause fitness loss [2,3,4,5,6,7,8]. The aforementioned outcomes of hybridization are largely dependent on the phylogenetic relatedness of parental species and/or the time of divergence from the most common ancestor. More closely related taxa, i.e., phylogenetically recent lineages, are expected to have viable hybrid offspring that could have adaptive advantages. The hybrid breakdown could be expected in the second or even in subsequent generations as a result of an accumulation of incompatibilities that produce reproductive barriers. On the other hand, hybridization between phylogenetically more divergent taxa could result in largely decreased viability or sterility of one or both sexes of F_1_ hybrids [6,9,10,11,12,13,14,15,16,17]. One of the hybridization consequences is asymmetrical gene flow resulting in introgression of mtDNA from one species to another, which could disturb mito-nuclear compatibility and potentially lead to substantial loss of hybrid fitness (e.g., [18,19,20]). There are several models that describe and explain the accumulation of genetic incompatibilities over time. For mito-nuclear incompatibilities, the Dobzhansky–Muller model was proposed as the most probable model of the evolution of incompatibilities (see [18] and references therein).

Phylogenetic relations within large-bodied newts (*Triturus*, Salamandridae) and a lack of complete reproductive barriers among species, with a wide range of hybridization outcomes, provide a substantial base for evolutionary studies of hybridization consequences. This monophyletic genus consists of two major groups, marbled and crested newts, which diverged from each other around 24 mya [21]. The marbled newts are *T. marmoratus* and *T. pygmeus*, while within crested newts, four groups are recognized based on genetic data and ecomorphological characteristics: (1) *T. ivanbureschi*, *T. anatolicus*, *T. karelinii*; (2) *T. carnifex, T. macedonicus*; (3) *T. cristatus*; and (4) *T. dobrogicus* [22]. The species have mostly parapatric distribution throughout Europe and parts of adjacent Asia. In the zones of species contact, interspecific hybridization occurs [23], resulting in dynamic hybrid zones in space and time. The movement of a hybrid zone implies advantages of one species at the expense of the other, which eventually leads to species spatial displacement. Most often, this scenario includes asymmetrical introgression of mtDNA from outcompeted to colonizing species [24,25,26,27,28,29,30,31]. Hybridization between *Triturus* species was also confirmed between autochthonous species and anthropogenically introduced species in areas well outside their natural range [32,33,34].

Natural hybrid populations could consist mostly of F_1_ hybrid generation, as in the case of two genetically well-separated species with different ecology and morphology, *T. cristatus* and *T. marmoratus,* which hybridize in western France (see [35] and references therein). These hybrids express typical heterosis; they are larger than the parental species and mostly sterile [36]. The opposite example is that of *T. ivanbureschi* × *T. macedonicus* hybrid populations on the Balkan Peninsula [27,37], which consist of an unknown generation of hybrid individuals derived from a long history of mutual hybrid crossings and backcrossing with both parental species [27,38,39]. *Triturus ivanbureschi* × *T. macedonicus* hybrids have intermediate body lengths related to parental species, and they are morphologically largely similar to both parental species [39]. In central Serbia, all populations of *T. macedonicus*, as well as *T. ivanbureschi* × *T. macedonicus* hybrids, have *T. ivanbureschi* mtDNA [25,26,27]. Asymmetrical mtDNA introgression is hypothesized to be a consequence of *T. macedonicus* range expansion over the range of *T. ivanbureschi* [25]. *Triturus ivanbureschi* and *T. macedonicus* are two moderately related, morphologically divergent species [22]. They diverge in body shape during postembryonic, larval development [40,41] as well as in the postmetamorphic period [42,43]. These species reproduce in similar aquatic habitats [44] but differ in reproductive traits, where *T. macedonicus* lay more eggs and have greater embryonic survival [43,45].

The mito-nuclear mismatch in *Triturus* populations in the central Balkans caused confusion in interpretations of species distribution and taxonomy (see [37] and references therein). In Serbia, many populations were considered to be *T. ivanbureschi* (syn. *T. karelinii*) according to mtDNA analysis, or *T. macedonicus* (syn. *T. carnifex*), according to morphology, were later confirmed as hybrid populations. Therefore, in previous studies, the reproductive data of some species are actually data for hybrid populations (Pirot [46,47]; Đurđevac [47]). These hybrid populations consist of *T. ivanbureschi* × *T. macedonicus* individuals of unknown generation (F_n_) with *T. ivanbureschi* mtDNA [27]. Body and egg sizes of hybrid females [46,47] were similar to those reported for parental species [43,46,48].

The first generation of *T. ivanbureschi* × *T. macedonicus* hybrids has not yet been confirmed in nature [27,38,39]. Since processes occurring in the F_1_ generation are of special interest for an explanation of forming and maintenance of hybrid zones [49], we set up experimental reciprocal crossings to obtain two types of hybrid genotypes: *T. ivanbureschi*-mothered hybrids (with *T. ivanbureschi* mtDNA, confirmed in natural populations) and *T. macedonicus*-mothered hybrids (with *T. macedonicus* mtDNA, not confirmed in natural populations). Our main goal was to explore the eventual differences in reproductive success of reciprocal hybrids carrying different parental mtDNA. Since growth and reproduction in newts are negatively interconnected due to resource allocation [50,51] and the period between the first and second reproductions is characterized by considerable growth [52], we followed life history traits in the first two consecutive years of reproduction. We recorded the life history traits directly related to reproductive output: body size (length and mass), the number of egg-laying females, duration of oviposition, the number and size of eggs and the number of hatchlings. As an estimation of reproductive success, we calculated the percentage of egg-laying females and the viability of their embryos. We also tested for eventual differences in reproductive success when hybrid females were bred with the hybrid males (with the same or reciprocal mtDNA) and with the males of parental species *T. ivanbureschi* and *T. macedonicus* (backcross combinations). Overall, we expected that hybrids with *T. macedonicus* mtDNA would have at least lower reproductive success, considering that *T. macedonicus* mtDNA is not present in natural populations. Failure in their reproductive success would indicate early exclusion of *T. macedonicus* mtDNA in the first generations of crossings upon *T. ivanbureschi* and *T. macedonicus* secondary contact.

## 2. Materials and Methods

Individuals of *T. ivanbureschi* and *T. macedonicus* were collected from natural populations away from their contact zones: *T. ivanbureschi* from Zli Dol, Serbia (42°25 N; 22°27 E) with permission obtained from the Serbian Ministry of Energy, Development and Environmental Protection (permit No. 353-01-75/2014-08) and *T. macedonicus* in Ceklin, Montenegro (42°21 N; 18°59 E) with permission obtained from the Agency for Environmental Protection, Montenegro (permit No. UPI-328/4). Genetic data confirm that these were *T. ivanbureschi* and *T. macedonicus* populations [27]. A series of crossbreeding experiments were carried out at the Institute for Biological Research “Siniša Stanković” and approved by the Ethics Committee of the Institute (decision Nos. 03-03/16 and 01-1949). By crossing parental species, two genotypes of hybrids (HI—with *T. ivanbureschi* mtDNA and HM—*T. macedonicus* mtDNA) were obtained in two different years:

- HI in 2016: *T. ivanbureschi* ♀ (*n* = 8) × *T. macedonicus* ♂ (*n* = 5);
- HM in 2017: *T. macedonicus* ♀ (*n* = 4) × *T. ivanbureschi* ♂ (*n* = 4).

Since growth and survival, as well as reproductive traits, can be affected by external factors (e.g., temperature, density, availability of food) and their mutual interactions (e.g., [53]), we kept the experimental settings and conditions the same throughout different years of the experiment. Crossing of parental species was done outdoors in 500 L plastic vats (separate vat for each breeding crossing). Plastic strips were provided for egg deposition. When females started laying eggs, they were transferred to the laboratory in separate 10 L aquariums half-filled with dechlorinated tap water. Eggs, embryos and larvae were raised in the same controlled experimental conditions in both years. The temperature was kept constant (18–19 °C) with a natural day/night regime. Eggs and embryos were raised in Petri dishes (up to 10 eggs/embryos per dish). Dechlorinated tap water was changed every other day. All Petri dishes were checked daily to remove non-developing eggs and arrested embryos. Larvae were raised in 2 L plastic containers (single larva per container). Containers were half-filled with dechlorinated tap water, which was changed every other day. Larvae were fed *ad libitum* with *Artemia* sp. and *Tubifex* sp. After metamorphosis, animals hibernated during winter in a cold chamber at a constant temperature (4 °C). During the first postmetamorphic year, between first and second hibernation, juveniles were kept in 200 L plastic vats placed outdoors with a similar number of animals per vat. Plastic vats were enriched with perforated bricks, providing shelter and plastic lids, which were used as floating platforms. Newts were fed *Tubifex* sp. and *Lumbricus* sp. twice a week.

**Figure 1:**
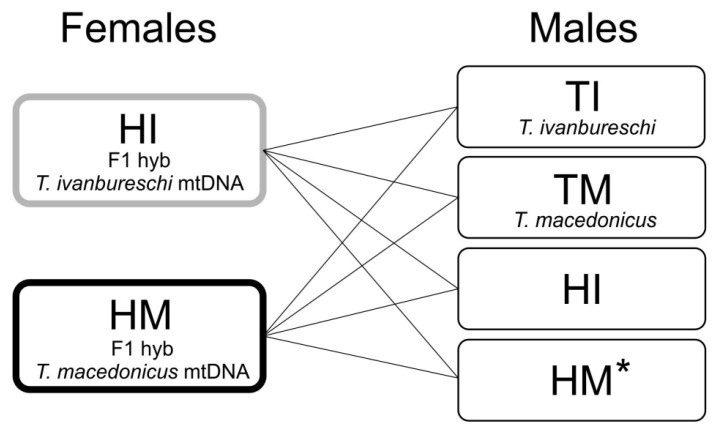
Schematic presentation of the experimental design. The F_1_ hybrid females with *T. ivanbureschi* mtDNA (HI) were crossed and backcrossed in 2018 and 2019. The F_1_ hybrid females with *T. macedonicus* mtDNA (HM) were crossed and backcrossed in 2019 and 2020. * Sexually mature HM males were available only in 2019, and therefore not involved in crossings with HI females in their first reproduction in 2018.

When hybrids had developed secondary sexual characters during the second postmetamorphic year, they were engaged in breeding series of mutual crossings and backcrossings with parental species (Figure 1). Animals hibernated before each breeding. HI females mated in 2018 (first reproduction) and 2019 (second reproduction). HM females mated in 2019 (first reproduction) and 2020 (second reproduction). The numbers of females involved in the breeding series were 23 HI and 20 HM in the first reproductive year and 25 HI and 20 HM in the second year of reproduction. To exclude the possibility of spermatozoid retention [54], the same females were crossed with the same males in two consecutive years. The only exceptions are two HI females, which were included in the crossing with HM males. Each crossing (see Figure 1) was conducted in a separate 200 L plastic container. Containers were set in the backyard of the Institute in semi-natural conditions. After females started laying eggs, they were transferred to separate 10 L aquariums, half-filled with dechlorinated water. Eggs and embryos were raised in the same controlled experimental conditions (see above). For more details of animals’ housing and experimental conditions, see [40,41,52].

Data for this study were collected during two consecutive years, in 2018 and 2019 for HI and in 2019 and 2020 for HM. To obtain measures of body size, females were photographed and weighed each year right after hibernation. Imaging was performed using a camera (Nikon D7100 or Sony DSC-F828) on a fixed stand with millimeter paper as a background. Body length (snout to vent length—SVL) was measured as the distance between the tip of the snout and the level of the posterior edge of the hind legs from the dorsal view using ImageJ software v. 1.50i [55]. Body mass (BM) was weighted to the nearest 0.01g using an electronic scale (MP300, Chyo Balance Corporation, Kyoto, Japan).

The eggs of newts consist of vitellus and a protective jelly layer. Females deposit individual eggs and wrap them in submerged vegetation [56], thus providing transparent plastic strips for egg deposition enabled easy observation of newly laid eggs. To estimate the number of laid eggs and egg size, we collected eggs on a daily basis and photographed them with a Nikon Digital Sight Fi2 camera attached to a Nikon SMZ800 stereo zoom microscope immediately after removal from the plastic strips for measurements. The number of eggs within each genotype was calculated from the first laid egg in common containers to the last laid egg in separate aquaria. Egg dimensions (maximum width of vitellus; maximum length and width of jelly) were taken using ImageJ software. Based on these measures, we calculated the volume of the vitellus and the volume of the entire egg. We calculated vitellus volume as the volume of a sphere: $Vv=4/3\times r^{3}\pi$, where r is the vitellus width/2. For the volume of egg, we measured ellipsoid volume: $Ve=4/3\times r_{1}r_{2}^{2}\pi ,$ where *r*_1_ and *r*_2_ are the radii length/2 and width of egg/2, respectively. The volume of the jelly was calculated as a difference between the volume of egg and the volume of vitellus: Vj=Ve−Vv. For statistical analysis of egg size, we used 100 eggs per genotype per year, which were taken randomly (RAND function in Microsoft Excel).

We also observed changes in other reproductive traits between the two reproductions, such as the number of egg-laying females, duration of oviposition, the total number of laid eggs and the total number of hatched larvae. Duration of oviposition was estimated as the number of days between the first and last laid egg within each genotype. To estimate reproductive success, we calculated the percentage of egg-laying females and the viability of their embryos. The percentage of egg-laying females was calculated as the ratio between the number of egg-laying females and the total number of females involved in breeding per hybrid genotype multiplied by 100. The viability of embryos was calculated as the ratio between the numbers of hatched larvae and the deposited eggs per hybrid genotype multiplied by 100.

Hybrid females of both genotypes were subdivided into four breeding groups exposed to different males (see Figure 1). As the exposure of females to different males can affect reproductive success in amphibians (e.g., [57]), we compared the percentages of egg-laying females and the viability of embryos separately within HI and HM females.

We used separated repeated measures ANOVAs to test for differences in the average body size (SVL and BM) and egg size (vitellus and jelly volumes), with female genotype as the categorical factor and year of reproduction as the within-subject (repeated measure) factor on the measured variables. Tukey’s HSD post hoc test was used to determine the statistical significance of between-group differences. Other reproductive traits (duration of oviposition, total number of eggs and total number of hatched larvae) were compared by the Kruskal–Wallis test since their values did not meet the criteria for ANOVA (assumptions of normality and sphericity). Differences in reproductive success were tested using differences between two proportions. Statistical analyses were done using Statistica10 software (StatSoft Inc., Tulsa, OK, USA, 2011).

## 3. Results

**Figure 2:**
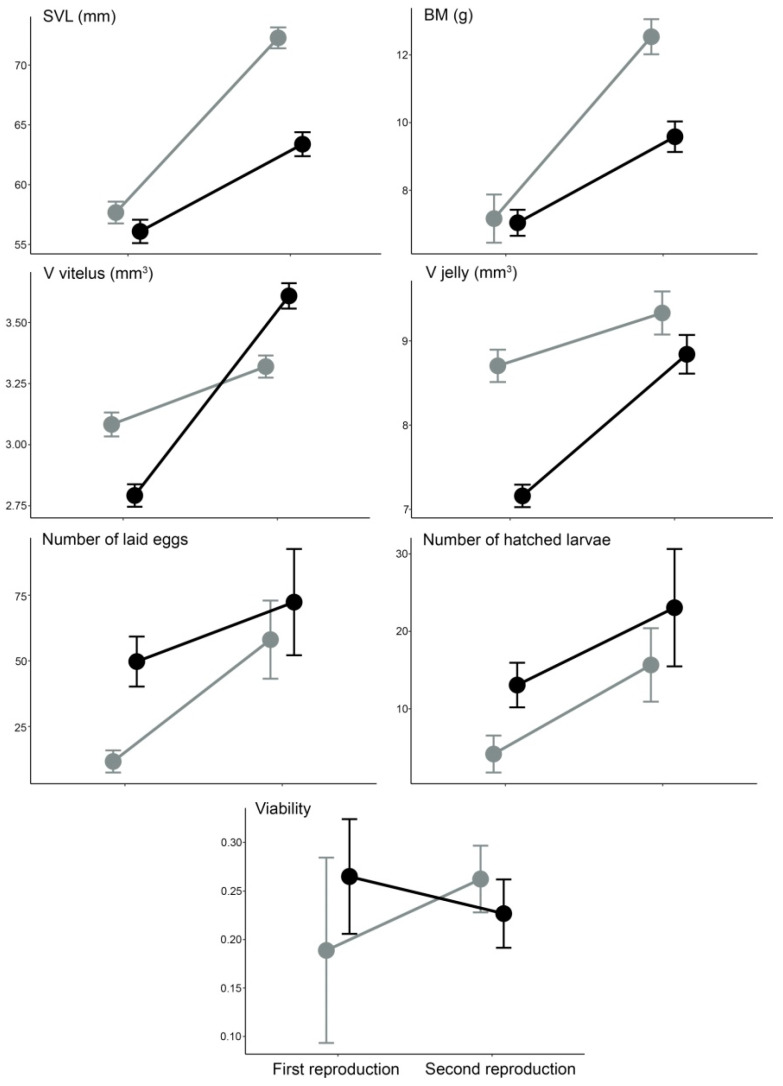
Ontogenetic differences in body size (length—SVL and mass—BM) and reproductive traits in the first two reproductive years of F1 hybrid females with *T. ivanbureschi* mtDNA, HI (gray) and with *T. macedonicus* mtDNA, HM (black). Values of each trait are represented as mean ± standard error per each genotype and year of reproduction.

The repeated measures ANOVA showed that reproductive year (SVL: F_1,38_ = 317.53, *p* < 0.0001; BM: F_1,38_ = 109.94, *p* < 0.0001), genotype (SVL: F_1,38_ = 15.34, *p* = 0.004; BM: F_1,38_ = 10.95, *p* = 0.002) and their interaction (SVL: F_1,38_ = 36.99, *p* < 0.0001; BM: F_1,38_ = 14.57, *p* = 0.0005) had a significant effect on the increase of body size between two reproductive years. Post-hoc tests showed a significant ontogenetic increase in body size (SVL and BM) within both genotypes. Differences in body size between genotypes were evident only in the second year of reproduction (Figure 2, Table 1).

For the volume of vitellus, repeated measures ANOVA (Figure 2) showed a significant effect of reproductive year (F_1,198_ = 62.10, *p* < 0.0001) and year × genotype interaction (F_1,198_ = 27.17, *p* < 0.0001), but non-significant effect of genotype alone (F_1,198_ = 0.08, *p* = 0.7714). Volume of jelly was significantly affected by both factors, reproductive year (F_1,198_ = 34.92, *p* < 0.0001) and genotype (F_1,198_ = 21.41, *p* < 0.0001), as well as their interaction (F_1,198_ = 7.28, *p* = 0.0076). Post-hoc tests revealed that there was no ontogenetic difference in the volume of vitellus and jelly within HI, while HM eggs were significantly larger in the second reproductive year. HI had larger eggs (volume of vitellus and jelly) than HM in the first reproductive year. In the second reproductive year, HM had a larger volume of vitellus but a similar jelly volume to HI (Figure 2, Table 1). Kruskal–Wallis analyses showed that there were no differences in other reproductive traits (duration of oviposition, total number of laid eggs and total number of hatched larvae) between and within hybrid genotypes (*p* > 0.05 in all comparisons, Figure 2).

**Figure 3:**
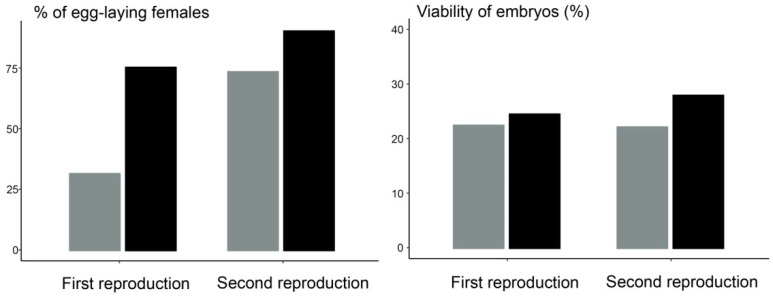
Differences in the percentage of egg-laying females and viability of embryos, as a measure of reproductive success during the first two reproductive years of F_1_ hybrid females with *T. ivanbureschi* mtDNA, HI (gray) and with *T. macedonicus* mtDNA, HM (black).

As the percentage of egg-laying females and viability of embryos also did not differ between HI and HM females in both reproductive years (*p* > 0.05 in all comparisons, Figure 3), we tested for differences in reproductive success between types of crossings (male effect) within each hybrid genotype. The percentage of egg-laying females did not differ within HI in both reproductive years regardless of different types of crossings, i.e., exposure to males of various genotypes. Within the HM females, the crossing of HM ♀ × ♂ had a relatively low percentage of egg-laying females, which was significantly lower from backcrossing of HM females with males of both parental species (*T. ivanbureschi* and *T. macedonicus*) in the first year, but this difference was lost in the second reproductive year (Table 2 and Table 3).

Offspring were obtained from all available breeding crossings in both reproductive years (see Figure 1). The viability of embryos obtained by different hybrid crossings and backcrossing was similar in the first reproductive year for both HI and HM females. For HI females, embryos obtained from the backcrossing of HI ♀ × *T. macedonicus* ♂ had greater viability than from the backcrossing of HI ♀ × *T. ivanbureschi* ♂. Embryos obtained from the reciprocal hybrid crossing (HI ♀ × HM ♂) had greater viability than those obtained from all other crossings of HI females and different male genotypes (HI, *T. ivanbureschi* and *T. macedonicus*) (Table 2 and Table 3). For HM females, embryos obtained from the crossing of HM ♀ × ♂ in the second reproductive year had the largest viability, significantly different from the crossing of reciprocal hybrids (HM ♀ × HI ♂), as well as from the backcrossing of HM females with *T. ivanbureschi* males (Table 2 and Table 3).

## 4. Discussion

In natural populations, overall reproductive output and success of F_1_ hybrid generation, particularly divergence of reciprocal hybrids, have an important impact on the long-term consequence of hybridization, as they can alter the direction of gene flow [58,59]. Therefore, the mito-nuclear incompatibility could shape patterns of genetic variation in hybrid zones and impact their dynamics. *Triturus* newts are a suitable model system for studies of mechanisms that lead to asymmetrical direction of mtDNA and introgression. Hybridization of the phylogenetically distant *T. cristatus* and *T. marmoratus* resulted in mtDNA introgression asymmetry due to divergence in the reproductive success of reciprocal crossings. More than 90% of individuals in natural populations have *T. cristatus* mtDNA [60], while F_2_ and further hybrid generations are rare [35,60]. Hybridization of *T. ivanbureschi* and *T. macedonicus*, two moderately related species, also resulted in an asymmetrical mtDNA introgression. *Triturus ivanbureschi* mtDNA is found across populations of *T. ivanbureschi, T. macedonicus* and *T. ivanbureschi* × *T. macedonicus,* in the Balkan Peninsula with the loss of *T. macedonicus* mtDNA in central Serbia [25,26,27].

Studies of *T. ivanbureschi* × *T. macedonicus* hybridization showed that hybrids have intermediate morphology compared to parental genotypes [39,40,41]. Hybridization affected physiological response resulting in raised oxidative stress parameters in hybrid larvae, which might have a negative impact on their survival [61,62,63]. However, fitness consequences of hybridization comprising slower growth rate, sex ratio disturbance and lower survival were not evident in F_1_ hybrids compared to maternal species during the early postmetamorphic period [52]. The morphological divergences between reciprocal hybrids were not recorded in previous morphological studies of larvae and recently metamorphosed juveniles. They have similar developmental patterns and growth rates [40,41].

We found that, at the beginning of the second postmetamorphic year, both F_1_ hybrids showed secondary sexual characters but did not diverge in body size. The divergence in size between reciprocal hybrids was recorded after the first reproduction, which coincides with the timing of sexual size dimorphism and divergence in size between hybrids and maternal species [52].

The other significant difference between reciprocal hybrids is related to egg size, which can have a profound impact on embryonic development and survival, as well as on larval growth and developmental rates [64]. Hybrids with *T. ivanbureschi* mtDNA laid larger eggs in the first reproduction (no differences between reciprocal hybrids in female body size), while eggs of hybrids with *T. macedonicus* mtDNA had substantially larger vitellus in the second reproduction, despite significantly smaller body size compared to hybrid females with *T. ivanbureschi* mtDNA. Additionally, reciprocal hybrids differed in the pattern of change in egg size between the first and second reproductions, where hybrids with *T. macedonicus* mtDNA showed a greater increase in vitellus size (see Figure 2). It is considered that the size of an amphibian egg is positively correlated with female body size. Thus, it is expected that older and larger females produce larger eggs, i.e., that ontogenetic changes of female body size should affect egg size [46,65,66]. Our results do not support previous notations. We suggest three mutually non-exclusive hypotheses that can be applied to explain the observed pattern of ontogenetic changes and divergences in vitellus size between reciprocal hybrids. First, the observed divergences may indicate that hybrids with *T. ivanbureschi* mtDNA invest more in somatic growth than in reproduction, while the opposite trend could be true for hybrids with *T. macedonicus* mtDNA, which could invest more in reproduction by enlarging vitellus volume. Second, our results could indicate that egg size is a heritable trait that is inherited by maternal investments through egg cytoplasm. In newts, egg size is a species-specific trait [47]. In a comparison of parental species, *T. macedonicus* has larger eggs than *T. ivanbureschi* [43]. The cytoplasm of the oocyte is full of maternal cytoplasmic components subsequently present in each cell of the new embryo [67], having an important role in the determination of egg size and possibly leading to significant divergences in egg size between two reciprocal hybrids. The third possible explanation for the divergence in egg size and female growth could be different sensitivity to external conditions of reciprocal hybrids. Previously, it was proposed that *T. macedonicus* is a thermophilic species [43,68]. Vitellogenesis occurs way before the breeding season, usually before hibernation [64], and it is dependent on different external and internal factors (e.g., [56]). It could be possible that external conditions were less suitable for F_1_ hybrids with *T. macedonicus* mtDNA at the time of their preparation for the first reproduction and vitellogenesis, i.e., hybrids with *T. macedonicus* mtDNA could be more susceptible to environmental variation during these periods. Studies on the plasticity of egg size and early life-history traits under different environmental conditions could give insight into the sensitivity of different genotypes to environmental conditions, with emphasis on different mtDNA groups. Hybrid females with both *T. ivanbureschi* and *T. macedonicus* mtDNA deposited a similar number of eggs in two reproductive years during a similar oviposition period. The number of eggs that hybrid females laid was in the range reported for both parental species in the same experimental settings [43], as well as for females from *T. ivanburechi* × *T. macedonicus* natural population [46], suggesting similar reproductive potential to that of the parental species. The observed pattern is opposite to *T. cristatus* and *T. marmoratus* hybridization, where hybrids have higher reproductive potential compared to parental species in experimental conditions, but low viability of eggs [69].

In our study, hybrid females were bred with four male genotypes (see Figure 1). In some amphibian taxa, females tend to choose males with whom they will engage in mating [56,57,70,71], which could affect overall reproductive success by decreasing or increasing the percentage of females that are involved in active breeding. Moreover, in hybridizing species, females’ choice could be a prezygotic barrier causing unidirectional mtDNA exchange if females of one species mate with males of the other and not vice versa [71]. Considering hybrid mutual mating and mating with parental species, it has been proposed that intermediate phenotypes can be disadvantageous for hybrid males, as they could be less attractive to females [7]. This could be true for newts, as it was shown that females do prefer larger males with a prominent crest [72]. In our experimental settings, the absence of differences between reciprocal hybrids in the percentage of egg-laying females, i.e., females engaged in active reproduction, suggests that females did not refuse to breed with either male genotype and their pickiness did not represent a barrier for either mtDNA to be passed on to the next generation. In comparisons within each female genotype, slight differences in percentages of egg-laying females in the first reproduction were not recorded in the second year of reproduction. Possibly, in their first reproduction, females preferred older (i.e., larger) males with prominent crests [72], as their conspecific males were of the same age or hybrid males were less successful in their first year of reproduction.

One characteristic of *Triturus* newts is that half of embryos die during development at the tail bud stage due to balanced lethal syndrome [73]. Viable eggs that had developed to hatchlings were obtained from all types of crossings (Figure 1) with no difference in embryo mortality between females with *T. ivanbureschi* and *T. macedonicus* mtDNA. However, viability differed in the second reproduction within reciprocal hybrid females when a different type of crossing (i.e., different male genotype) was included. Differences in the viability of embryos from different crossings and backcrossings indicate a possible decrease in survival of the F_2_ generation. For example, the backcrossing of hybrid females with *T. ivanbureschi* mtDNA and *T. macedonicus* was more successful than the one including *T. ivanbureschi* males. The obtained result could back up the hypothesis of postglacial species displacement and *T. macedonicus* nuclear DNA spreading [25]. This hypothesis suggests that, throughout generations, subsequent backcrossing of hybrid females with *T. ivanbuerschi* mtDNA with males of *T. macedonicus* diminished *T. ivanbureschi* nuclear DNA, rebuilt *T. macedonicus* nuclear DNA and retained *T. ivanbureschi* mtDNA [25]. The difference in the viability of F_2_ generations should be confirmed and further investigated in a larger study that would include postembryonic developmental stages, as it was shown that asymmetry in the frequency of reciprocal hybrids emerged after embryonic development in *T. cristatus* × *T. marmoratus* hybridization [59].

## 5. Conclusions

We found that the reproductive traits and success of reciprocal *T. ivanbureschi* × *T. macedonicus* F_1_ hybrids (with *T. ivanbureschi* or *T. macedonicus* mtDNA) is largely similar, expressing some differences depending on trait, year of reproduction and male genotype involved in mating. Overall, the obtained results suggest that the loss of *T. macedonicus* mtDNA emerged in F_2_ or subsequent hybrid generations. Major cito-nuclear incompatibilities are often masked in the first generation and manifested in F_2_ or subsequent hybrid generations due to their accumulation, the phenomenon known as hybrid breakdown (e.g., [10,16]). The viability of F_2_ embryos, obtained from mutual hybrid crossings and backcrossing with both parental species, points out a possible decrease of fitness in the F_2_ generation. However, these results are only preliminary and should be tested further.

Females were monitored in the first two years of reproduction, so possibly results considering success in crossings with different male genotypes could change with the age of females. The postembryonic viability of F_2_ generation should be analyzed, as possible differences in survival can occur during larval development, metamorphosis or even during the postmetamorphic period. An experiment, which would include females and males of various ages of *T. ivanbureschi*, *T. macedonicus* and hybrids with both types of mtDNA, would enable females to choose between different male genotypes. The suggested experimental design could give another insight into processes that took place a long time ago, upon the initial secondary contact between two moderately related species of *T. ivanbureschi* and *T. macedonicus.*

## Figures and Tables

**Table 1:** Differences in body size and egg volume between F1 hybrid females with *T. ivanbureschi* mtDNA (HI) and *T. macedonicus* mtDNA (HM) during the first two reproductive years. Analyzed traits: SVL—snout to vent length, BM—body mass, Vv—vitellus volume, Vj—jelly volume. Level of significances of pairwise comparisons: ns = not significant.

Comparisons	Females		Eggs	
	SVL	BM	Vv	Vj
within genotypes (I vs. II year)				
HI	0.0002	0.0002	ns	ns
HM	0.0002	0.0004	<0.0001	<0.0001
between genotypes (HI vs. HM)				
I year	ns	ns	0.0011	<0.0001
II year	0.0002	0.0002	0.0051	ns

**Table 2:** Reproductive success (percentages of egg-laying females and viability of embryos) of F_1_ hybrid females with *T. ivanburechi* mtDNA (HI) and *T. macedonicus* mtDNA (HM) exposed to different males (see Figure 1 for various breeding crossings and abbreviations). Sexually mature HM males were not available in 2018 and therefore not involved in crossings with HI females in their first reproductive year.

Breeding Crossings (♀ × ♂)	Egg-Laying Females (%)		Viability (%)	
	I Year	II Year	I Year	II Year
HI × HI	22	43	26	20
HI × HM	/	50	/	35
HI × TI	14	100	8	18
HI × TM	57	100	33	25
HM × HM	20	100	19	35
HM × HI	80	100	20	23
HM × TI	100	100	26	26
HM × TM	100	60	23	27

**Table 3:** Pairwise comparison of reproductive success (percentages of egg-laying females and viability of embryos) of F_1_ hybrid females with *T. ivanbureschi* mtDNA (HI) and *T. macedonicus* mtDNA (HM) exposed to different males (see Figure 1 for various breeding crossings and abbreviations). The comparisons were done within HI and HM females separately. Sexually mature HM males were not available in 2018 and therefore not involved in crossings with HI females in their first reproductive year. Level of significances of pairwise comparisons: ns = not significant.

Compared Breeding Crossings (♀ × ♂)	Egg-Laying Females (%)		Viability (%)	
	I Year	II Year	I Year	II Year
HI × HI vs. HI × TI	ns	ns	ns	ns
HI × HI vs. HI × TM	ns	ns	ns	ns
HI × TI vs. HI × TM	ns	ns	ns	0.0310
HI × HI vs. HI × HM	/	ns	/	0.0002
HI × TI vs. HI × HM	/	ns	/	<0.0001
HI × TM vs. HI × HM	/	ns	/	0.0140
HM × HM vs. HM × TI	0.0320	ns	ns	<0.0001
HM × HM vs. HM × TM	0.0320	ns	ns	ns
HM × TI vs. HM × TM	ns	ns	ns	ns
HM × HM vs. HM × HI	ns	ns	ns	0.0160
HM × TI vs. HM × HM	ns	ns	ns	ns
HI × HI vs. HM × HM	ns	ns	ns	ns

## Data Availability

Data is available upon request from the corresponding author.
